# Mechanisms of indigo naturalis on treating ulcerative colitis explored by GEO gene chips combined with network pharmacology and molecular docking

**DOI:** 10.1038/s41598-020-71030-w

**Published:** 2020-09-16

**Authors:** Sizhen Gu, Yan Xue, Yang Gao, Shuyang Shen, Yuli Zhang, Kanjun Chen, Shigui Xue, Ji Pan, Yini Tang, Hui Zhu, Huan Wu, Danbo Dou

**Affiliations:** 1 Traditional Chinese Medicine Department, Shuguang Hospital Affiliated to Shanghai University of Traditional Chinese Medicine, 528 Zhang Heng Road, Pudong New area, Shanghai, 201203 China; 2 Shi’s Center of Orthopedics and Traumatology, Shuguang Hospital Affiliated to Shanghai University of Traditional Chinese Medicine, Shanghai, 201203 China; 3 Digestive Endoscopy Center, Shuguang Hospital Affiliated to Shanghai University of Traditional Chinese Medicine, Shanghai, 201203 China; 4 Emergency Department, Shuguang Hospital Affiliated to Shanghai University of Traditional Chinese Medicine, Shanghai, 201203 China

**Keywords:** Inflammatory bowel disease, High-throughput screening, Network topology

## Abstract

Oral administration of indigo naturalis (IN) can induce remission in ulcerative colitis (UC); however, the underlying mechanism remains unknown. The main active components and targets of IN were obtained by searching three traditional Chinese medicine network databases such as TCMSP and five Targets fishing databases such as PharmMapper. UC disease targets were obtained from three disease databases such as DrugBank,combined with four GEO gene chips. IN-UC targets were identified by matching the two. A protein–protein interaction network was constructed, and the core targets were screened according to the topological structure. GO and KEGG enrichment analysis and bioGPS localization were performed,and an Herbs-Components-Targets network, a Compound Targets-Organs location network, and a Core Targets-Signal Pathways network were established. Molecular docking technology was used to verify the main compounds-targets. Ten core active components and 184 compound targets of IN-UC, of which 43 were core targets, were enriched and analyzed by bioGPS, GO, and KEGG. The therapeutic effect of IN on UC may involve activation of systemic immunity, which is involved in the regulation of nuclear transcription, protein phosphorylation, cytokine activity, reactive oxygen metabolism, epithelial cell proliferation, and cell apoptosis through Th17 cell differentiation, the Jak-STAT and IL-17 signaling pathways, toll-like and NOD-like receptors, and other cellular and innate immune signaling pathways. The molecular mechanism underlying the effect of IN on inducing UC remission was predicted using a network pharmacology method, thereby providing a theoretical basis for further study of the effective components and mechanism of IN in the treatment of UC.

## Introduction

Ulcerative colitis (UC) is a refractory intestinal disease with alternating onset and remission and a long disease course, which seriously affects the health and quality of life of patients. The goal of treatment is to control clinical symptoms, induce and maintain remission, promote mucosal healing, and reduce recurrence^1^. The current treatment of UC consists of 5-aminosalicylic acid, steroids, immunosuppressants, and biological agents. However, dependence on these drugs, drug resistance, intolerance, loss of response, and opportunistic infection are increasingly becoming clinical problems. Furthermore, clinical trials have shown unsatisfactory clinical response rates and clinical remission rates for these drugs. Therefore, many patients with inflammatory bowel disease (IBD) as well as physicians and researchers are increasingly considering complementary and alternative medicine (CAM) options^2,3^. In North American and European studies, the rate of current or past use of CAM for the treatment of IBD is 21%–60%^4,5^, of which herbal medicine, especially CAM intervention, is the first choice^6^.

In recent years, Japanese scholars have actively explored single herbal treatment of UC and found that oral administration of indigo naturalis (IN) can effectively treat intractable cases of mucosal aplastic disorders. UC remission was induced using the traditional Chinese medicine compound Xi lei san enema^7,8^. IN ointment has been used externally for the treatment of psoriasis^9^. A series of single-center, open, prospective, single-arm clinical trials and national multicenter, prospective, double-blind, randomized controlled trials were conducted successively. Assessment of the clinical outcomes at 8 weeks confirmed the safety and efficacy of IN administered orally for the treatment of UC. The clinical remission rate (Mayo score 2, no score > 1) and mucosal healing rate (Mayo endoscopy score 1) of the IN group are higher than those of the placebo group. Even in hormone-dependent or anti-TNF-α refractory patients, the clinical response rate (Mayo score decreased by 3 points, at least 30% lower than the baseline, rectal bleeding score or absolute rectal bleeding score decreased by at least 1 point) is very high, and such results are very encouraging.

As a supplementary alternative medicine, traditional Chinese medicine has a rich history and has shown good results in the treatment of UC. Previous studies from our group showed that oral Chinese medicine compounds were effective for the treatment of UC, inducing and maintaining remission, although the time until the effect was evident was relatively slow^10–12^.

Compared with western medicine, traditional Chinese medicine decoction may not quickly induce UC remission and control clinical symptoms. In addition, because of the quality of herbal medicine and other factors, the curative effect of traditional Chinese medicine is not stable enough. The patients reported a poor taste of traditional Chinese medicine decoction and had poor follow-up compliance.

Based on a series of studies in Japan, our team analyzed traditional Chinese medicine compound prescriptions containing IN in the domestic periodical databases (CNKI, VIP, Wanfang), and identified 43 prescribed traditional Chinese medicine compound prescriptions, including 37 enema prescriptions, four oral prescriptions, one rectal drip prescription, and two endoscopic spray prescriptions. These results indicate that IN is mainly used externally for the treatment of UC, whereas there are few oral prescriptions. At present, the only oral proprietary Chinese medicine developed for the treatment of UC containing IN is Five Sophora flavescens enteric-coated capsule (FSEC) (containing Qingdai, Sophora flavescens, Bletilla striata, Ulmus pumila and licorice). Phase II and III clinical trials show that the efficacy of FSEC in the treatment of UC is not inferior to that of mesalazine granules and enteric-coated tablets^13,14^. Oral administration of compound and single IN in capsule form not only has a good curative effect, but also prevents problems such as poor taste and poor compliance associated with traditional Chinese medicine decoctions.

**Figure 1:**
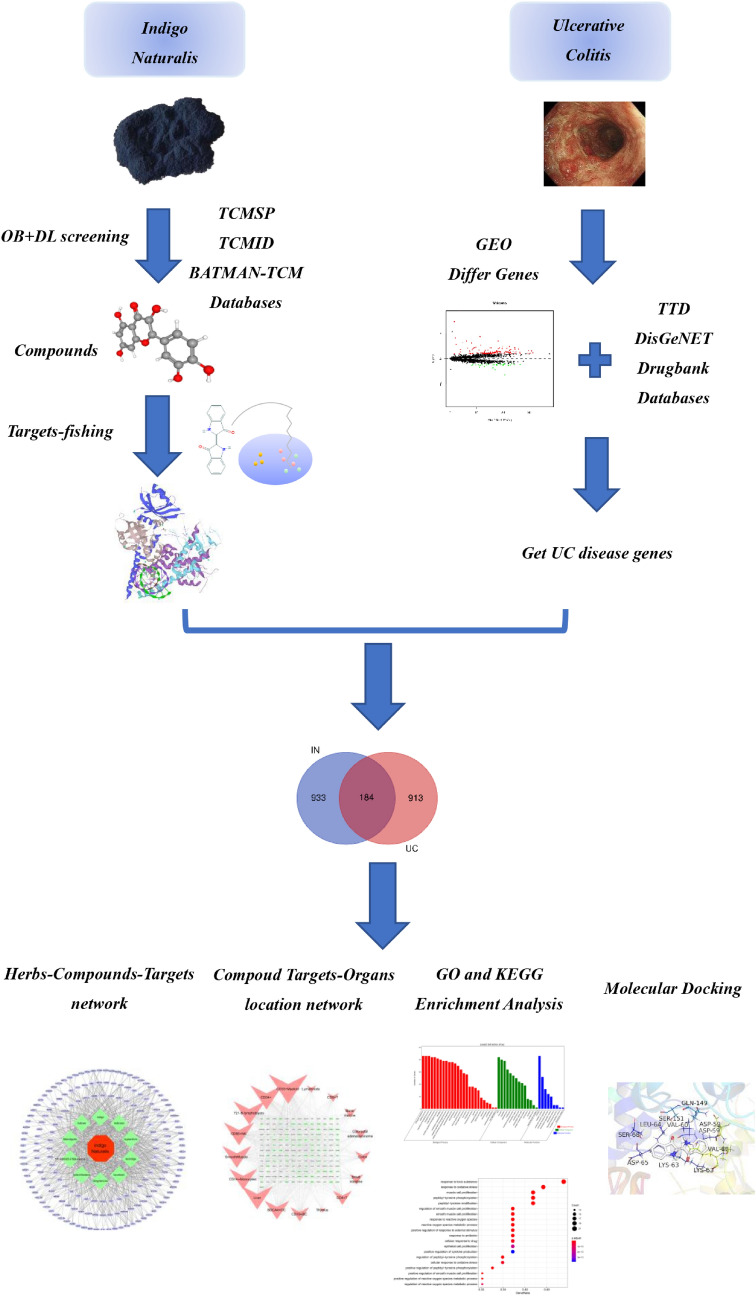
Framework based on an integration strategy of network pharmacology.

Under the premise of clinical effectiveness, we need to deeply investigate the mechanism of IN. The mechanism underlying the effect of IN on inducing UC mucosal healing is not clear^1,15^. In this study, we used the emerging traditional Chinese medicine network pharmacology method to predict the targets of IN, and systematically predict the mechanism of action of IN in UC. The method used in the study is as follows: a search of multiple traditional Chinese medicine network databases was performed to identify the main and essential active components and targets of IN at the same time, we searched multiple disease databases in combination with the results of multiple GEO gene chips to obtain UC disease targets, and matched them to obtain IN-UC compound targets. A protein–protein interaction network was constructed, and core targets were screened according to topological structure. GO and KEGG enrichment analyses were performed to construct Herbs-Components-Targets networks, Compound Targets-Organs location networks, and Core Targets-Signal Pathways networks. Finally, molecular docking technology was used to verify the main compounds-targets. An outline of the method is shown in Fig. 1.

## Materials and methods

### Construction of a database of IN main active ingredients

The traditional Chinese medicine system pharmacology database and analysis platform (TCMSP, https://tcmspw.com/tcmsp.php)^16^, the bioinformatics analysis tool for the molecular mechanism of traditional Chinese medicine (BATMAN-TCM, https://bionet.ncpsb.org/batman-tcm/index.php/Home/Index/index)^17^, and the comprehensive database of traditional Chinese Medicine (TCMID, https://119.3.41.228:8000/tcmid/)^18^ were used to identify the active ingredients of IN. The main active ingredients were then selected according to the optimal toxicokinetic ADME rules reported in the literature (OB ≥ 30% dl ≥ 0.18)^19^. If the compounds did not meet the screening criteria, they were included if they were reported as effective against UC in the relevant literature^20–22^. The related compounds were input into PubChem (https://pubchem.ncbi.nlm.nih.gov/) and Pharmgkb (https://www.pharmgkb.org/) to obtain the molecular structure of the related compounds.

### Potential targets of IN

The active components of a drug perform related biological functions through the relevant targets. In addition to obtaining the targets of the core active ingredients of indigo directly from the TCMSP database, information on the small molecule structure of the core active ingredient (Canonical SMILES) was used for target identification. Similarity ensemble approach (https://sea.bkslab.org/)^23^, STITCH (https://stitch.embl.de/)^24^ and Swiss Target Prediction (https://new.swisstargetprediction.ch/)^25^, and PharmMapper (https://lilabecust.cn/pharmmapper/)^26^ identified a large number of possible targets for IN.

### Construction of a UC-related targets database

First, microarray data of differentially expressed mRNAs in the intestinal mucosa between the normal group and the UC group were obtained from the GEO database (https://www.ncbi.nlm.nih.gov/geo/), Series: GSE87466, GSE65114, GSE9686, GSE10616. Sva and Limma of Rmur3.6.1 were used to carry out joint analysis of multiple chips and correct data batches. The two software packages can be obtained from (https://www.bioconductor.org/). Genes with an adjusted *P* < 0.05 and log_2_(fold change) > 1 or log_2_(fold change) <  − 1 were considered significantly differentially expressed and UC-related targets. In addition, UC-related disease targets were integrated in the database, including the: DrugBank database (https://www.drugbank.ca/)^27^, TTD database (https://db.idrblab.org/ttd/)^28^, and DisGeNET database (https://www.disgenet.org/web/DisGeNET/menu/home)^29^, using “Ulcerative Colitis” as the keyword, combined with the Uniprot database (https://www.uniprot.org/)^30^, and GEO analysis results to eliminate repeated disease targets and establish the disease target database of UC.

### Construction of the PPI interaction network

Based the above analyses, the core active ingredient target of indigo was matched with the disease target of UC to obtain the compound target of IN-UC. The VENN map was drawn by Bioinformatics (https://bioinformatics.psb.ugent.be/webtools/Venn/) and the PPI network of the target was obtained by using the String online tool (https://string-db.org/)^31^.

### Construction of an “Herbs-Components-Targets” network of IN

Based on the PPI network obtained above, the “Herbs-Components-Targets” network (H-C-T network) of IN was constructed using Cytoscape3.6.1(https://www.cytoscape.org/)^32^. According to the topological characteristics of the network, the three most important parameters were selected to screen the core composite targets of Indigo: degree of Degree Centrality (DC)^33^, Closeness Centrality (CC)^34^, and Betweenness Centrality (BC)^35^. Degree centrality refers to the number of other nodes associated with a node in the network. The greater the degree centrality, the greater the importance of the node. Betweenness centrality calculates the number of shortest paths through a node. The more the number of shortest paths through a node, the higher its intermediary centrality. Closeness centrality calculates the sum of the distances from one point to all other points. The smaller the sum, the shorter the path from this point to all other points, which means that the point is closer to all other points. The levels of these three parameters represented the topological importance of the nodes in the network, they can reflect the role and influence of the corresponding nodes in the whole network and importance of the nodes was positively correlated with the output value in the network. According to relevant literature reports, the target showing two-fold the median value was selected for DC^36^, and the target with median value for BC and CC was selected^37^ to obtain a more accurate core targets.

### Construction of a compoud targets-organs location network

The metabolism of IN in vivo is not clear, and multiple tissues and organs may be involved in the intestinal mucosal repair of UC induced by IN. Therefore, the BioGPS database (https://biogps.org) was used to examine the mRNA expression profile of each IN-UC compound target at the organ tissue level^38^. The database provides gene expression data obtained by direct measurement of gene expression by microarray analysis^39^. The specific steps are as follows: first, the distribution data of mRNA expression of each compound target in 84 organs and tissues are obtained, and then the average value of each mRNA in each tissue and the overall average value in all tissues are calculated. Finally, the related organs and tissues whose mRNA expression is higher than the overall average are extracted^40^, and the Compoud Targets-Organs location network is established by Cytoscape 3.6.1.

### GO and KEGG enrichment analysis

After obtaining the core target, we used ClusterProfiler^41^ GO and clusterProfiler KEGG of R 3.6.1 to analyze the GO and KEGG enrichment of the core target. The two software packages can be obtained from (https://www.bioconductor.org/) acquisition. GO enrichment mainly analyzes the biological process, cellular composition, and molecular function of the target, whereas KEEG (www.kegg.jp/kegg/kegg1.html) enrichment analyzes the potential biological pathways and functions associated with the target.

### Active components-targets docking

Three components were selected among the core components of IN and docked with three proteins selected from the core targets to verify the accuracy of the main components and prediction targets. The candidate composition and the target crystal structure were downloaded from the TCMSP database and RCSB protein data (https://www.pdb.org/), respectively. The latter preferably selects a model with ligand binding smaller than 3 Å, and then imports the crystal into the Pymol 1.7.2.1 Software (https://pymol.org/2/) for dehydration, hydrogenation, and separation of ligands; it then imports AutoDockTools 1.5.6 to construct the docking grid box^42,43^ for each target**.** Docking was completed by Autodock Vina 1.1.2 software^44^, and the molecules with the lowest binding energy in the docking conformation were selected to observe the binding effect by matching with the original ligands and intermolecular interactions (such as hydrophobicity, cation-π, anion-π, π-π stacking, hydrogen bonding, etc.).

## Results

### Targets prediction and analysis of IN

**Figure 2:**
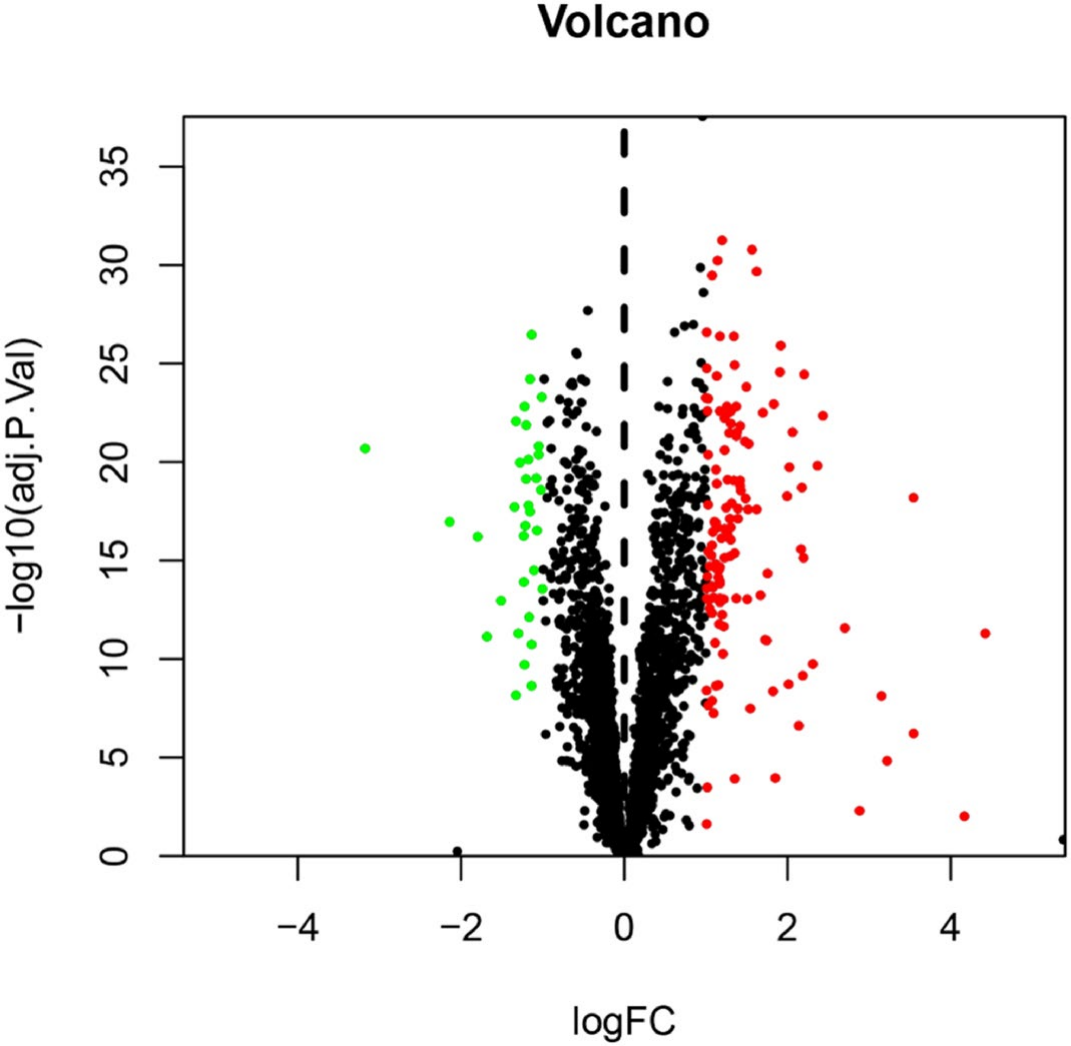
Differential genes volcano map jointly analyzed by four GEO chips. mRNA of intestinal mucosal biopsies from normal group and UC group.

**Figure 3:**
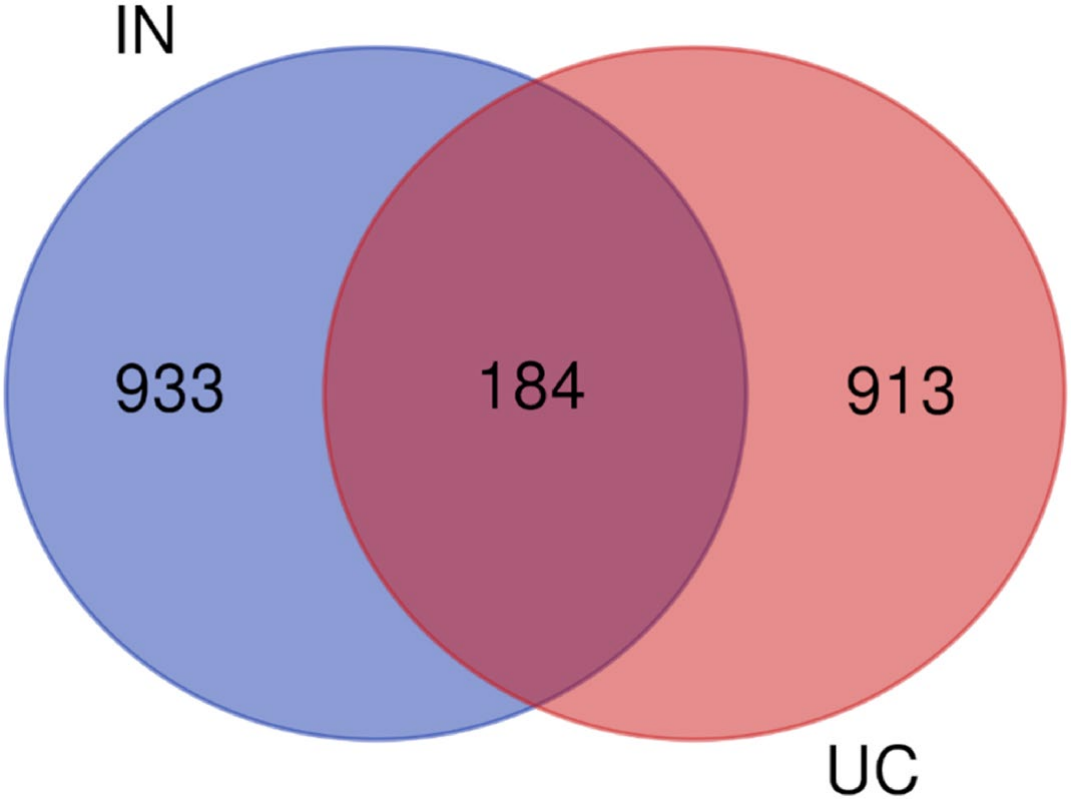
Venn diagram of the targets in UC and IN.

A total of 29 active ingredients were obtained from TSMSP, BATMAN-TCM, and TCMID, and nine core active components were selected according to the screening criteria of ADME OB ≥ 30% and DL ≥ 0.18. However, tryptanthrin was the third most effective component of indigo, and the related literature confirmed that it showed protective effects in an experimental UC animal model^21,22^. Therefore, it was included although it did not meet the OB and DL criteria. Finally, ten core active components (Table 1) were included. According to the Canonical SMILES number of core active ingredients of IN, 933 indigo targets were identified by target fishing and by integrating the data obtained from TCMSP, SEA, STITCH, STP, and PM databases (Supplementary Table S1). Joint analysis of four gene chips in the GEO database (GSE87466,GSE65114, GSE10616, GSE9686) identified 165 differentially expressed genes related to UC (Supplementary Table S2), which were used to build a volcano map (Fig. 2). In addition, we integrated DrugBank, DisGeNET, and TTD database disease targets and combined them with the GEO results to eliminate duplicates, resulting in the identification of 913 disease targets (Supplementary Table S3). The core active component targets of indigo chinensis were matched with the disease targets of UC, resulting in the selection of 184 composite targets of indigo and UC (Fig. 3) (Supplementary Table S4).

**Table 1:**
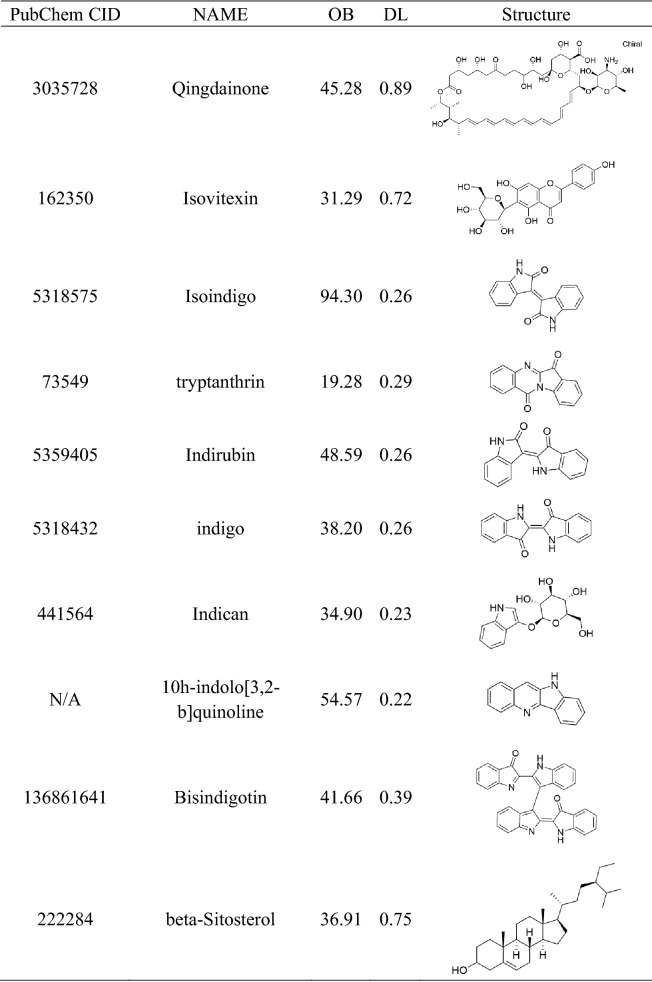
Core components of IN.

### “Herbs-Components-Targets” network of IN analysis

**Figure 4:**
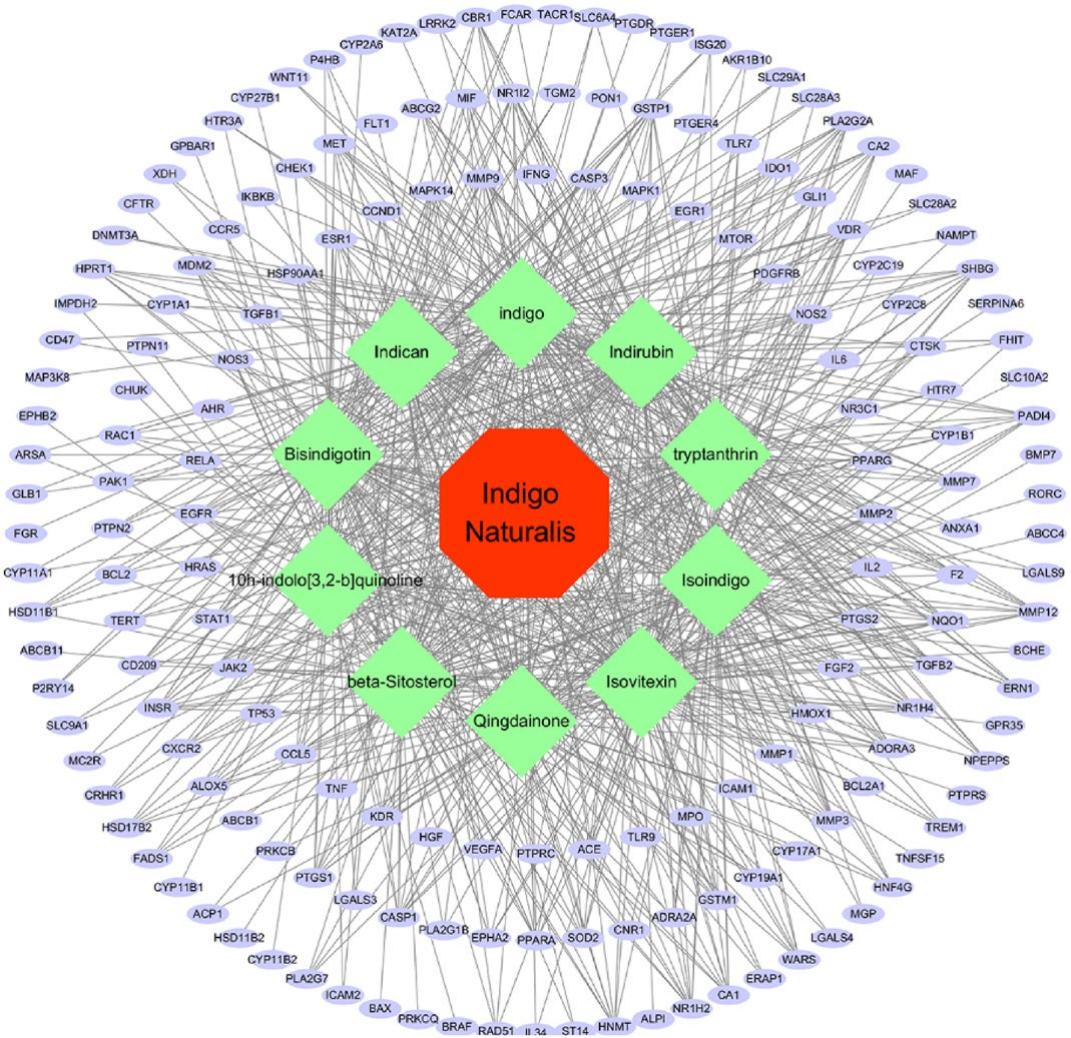
Herb-ingredients-targets (H-I-T) network. Red node represents IN, green nodes represent core active compounds of IN, purple nodes represent targets of IN.

**Figure 5:**
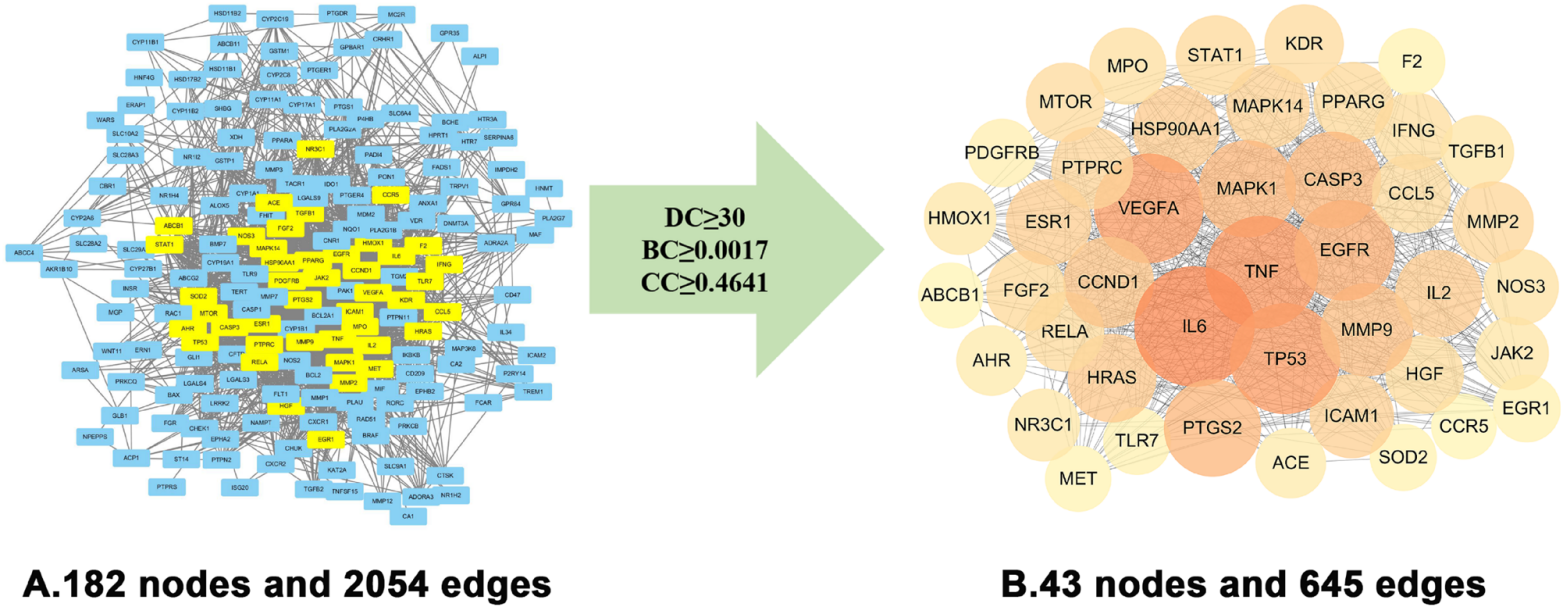
The process of topological screening for the PPI network. The PPI network diagram of 43 core targets was obtained by screening 182 IN-UC composite targets through DC,BC,CC.

**Figure 6:**
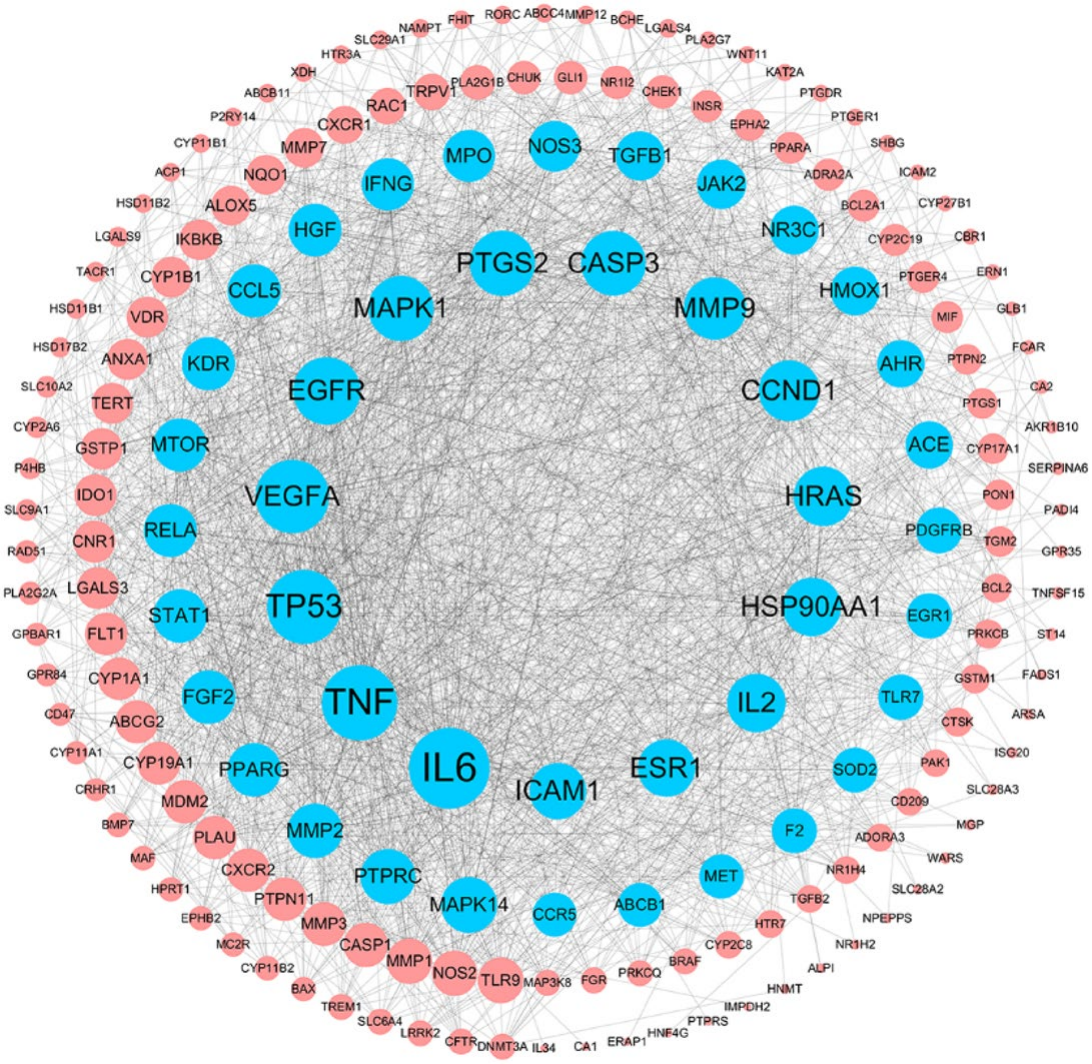
The PPI network of 182 nodes. The node size is proportional to the target degree in the network. The Blue nodes are the core targets of IN-UC.

The IN-UC composite targets identified were input into STRING to remove the unconnected target, and the PPI network was obtained. Cytoscape 3.6.1, showed that the network contained 182 nodes and 2054 edges. The “Herbs-Components-Targets” network of IN was constructed (Fig. 4), including 192 nodes and 748 edges. Then, according to the characteristics of the network topology, DC selects the target showing twofold the median value^36^, and BC and CC select the target with the median value^37^. The median DC of the network was 15 DC BC and the median value of CC was 0.0017 and 0.4641, respectively. After network analysis with NetworkAnalyzer, 43 core targets were selected (see Fig. 5 for the process of screening core target network in Table 2 and the PPI network, including 43 nodes and 645 edges). At the same time, we constructed the network diagram of core targets and non-core targets (Fig. 6).

**Table 2:** Information on 43 core targets.

Uniprot ID	Gene symbol	Protein name	Degree
P05231	IL6	Interleukin-6	106
P01375	TNF	Tumor necrosis factor	95
P04637	TP53	Cellular tumor antigen p53	92
P15692	VEGFA	Vascular endothelial growth factor A	91
P00533	EGFR	Epidermal growth factor receptor	79
P35354	PTGS2	Prostaglandin G/H synthase 2	74
P28482	MAPK1	Mitogen-activated protein kinase 1	74
P42574	CASP3	Caspase-3	73
P14780	MMP9	Matrix metalloproteinase-9	68
P24385	CCND1	G1/S-specific cyclin-D1	65
P01112	HRAS	GTPase HRas	62
P07900	HSP90AA1	Heat shock protein HSP 90-alpha	60
P60568	IL2	Interleukin-2	60
P03372	ESR1	Estrogen receptor	58
P05362	ICAM1	Intercellular adhesion molecule 1	56
Q16539	MAPK14	Mitogen-activated protein kinase 14	55
P08575	PTPRC	Receptor-type tyrosine-protein phosphatase C	53
P08253	MMP2	72 kDa type IV collagenase	52
P42224	STAT1	Signal transducer and activator of transcription 1-alpha/beta	50
P37231	PPARG	Peroxisome proliferator-activated receptor gamma	50
P09038	FGF2	Fibroblast growth factor 2	50
P35968	KDR	Vascular endothelial growth factor receptor 2	49
P13501	CCL5	C–C motif chemokine 5	49
Q04206	RELA	Transcription factor p65	49
P42345	MTOR	Serine/threonine-protein kinase mTOR	49
Q59H59	HGF	Hepatocyte growth factor isoform 1 preproprotein variant	48
P01579	IFNG	Interferon gamma	47
P05164	MPO	Myeloperoxidase	46
P29474	NOS3	Nitric oxide synthase	44
O60674	JAK2	Tyrosine-protein kinase JAK2	41
P01137	TGFB1	Transforming growth factor beta-1 proprotein	41
P04150	NR3C1	Glucocorticoid receptor	40
P09601	HMOX1	Heme oxygenase 1	40
P35869	AHR	Aryl hydrocarbon receptor	37
Q9BYF1	ACE	Angiotensin-converting enzyme 2	37
P09619	PDGFRB	Platelet-derived growth factor receptor beta	34
P18146	EGR1	Early growth response protein 1	33
Q9NYK1	TLR7	Toll-like receptor 7	33
P04179	SOD2	Superoxide dismutase [Mn]	32
Q86WA1	F2	coagulation factor II	32
P08581	MET	Hepatocyte growth factor receptor	31
P51681	CCR5	C–C chemokine receptor type 5	30
P08183	ABCB1	ATP-dependent translocase ABCB1	30

### Compoud targets-organs location network analysis

**Figure 7:**
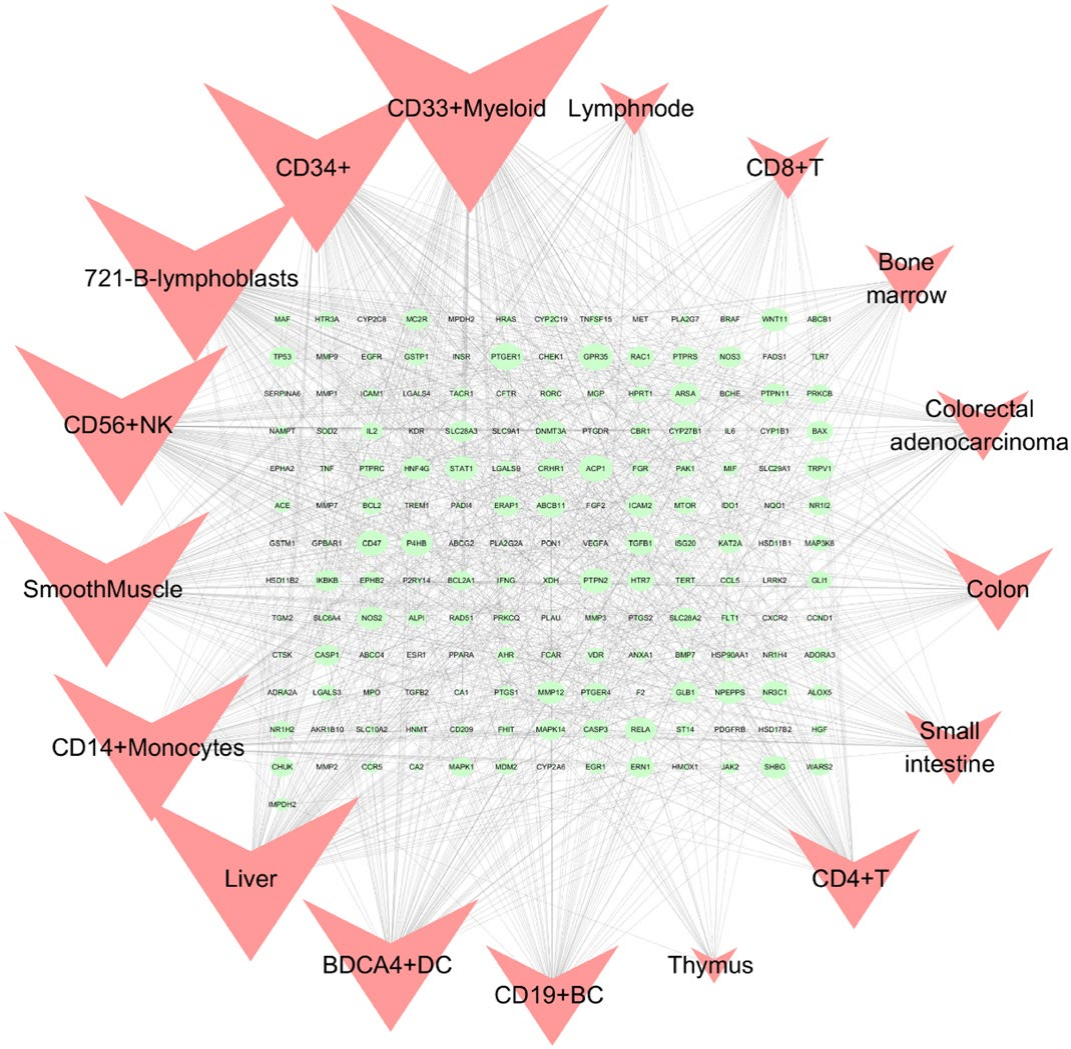
Gene expression data were based on gene expression microarray analysis results in BioGPS. Targets-organs location network (H–O): Nodes represent targets and organ locations. Node size is relative to degree.

Two of the target proteins, GPR84 and TLR9, did not have mRNA expression profiles in BioGPS. We analyzed the mRNA expression profiles of 180 compound targets, of which 170 genes showed higher-than-average mRNA expression in 17 UC-related immune organs, including colon (45 targets), small intestine (39 targets), liver (88 targets), colorectal adenocarcinoma (36 targets), smooth muscle (83 targets), bone marrow (35 targets), thymus (18 targets), lymph node (27 targets), CD33 + myeloid (110 targets), CD34 + (90 targets), 721-B-lymphoblasts (87 targets), CD56 + NK (85 targets), CD14 + monocytes (77 targets), CD4 + T (52 targets), CD8 + T (32 targets), BDCA4 + DC (69 targets), and CD19 + BC (52 targets) (Supplementary Table S5, S6). Then, the Targets-Organs Location Network (Fig. 7) was constructed, which contained 186 nodes and 997 edges. Most of the targets were highly expressed in several organs and tissues simultaneously, indicating that these organs are closely related to the targets of indigo. In addition, the 17 organs are highly related to immunity, suggesting that the therapeutic effect of IN on UC may involve activation of systemic immunity.

### GO and KEGG enrichment analysis

**Figure 8:**
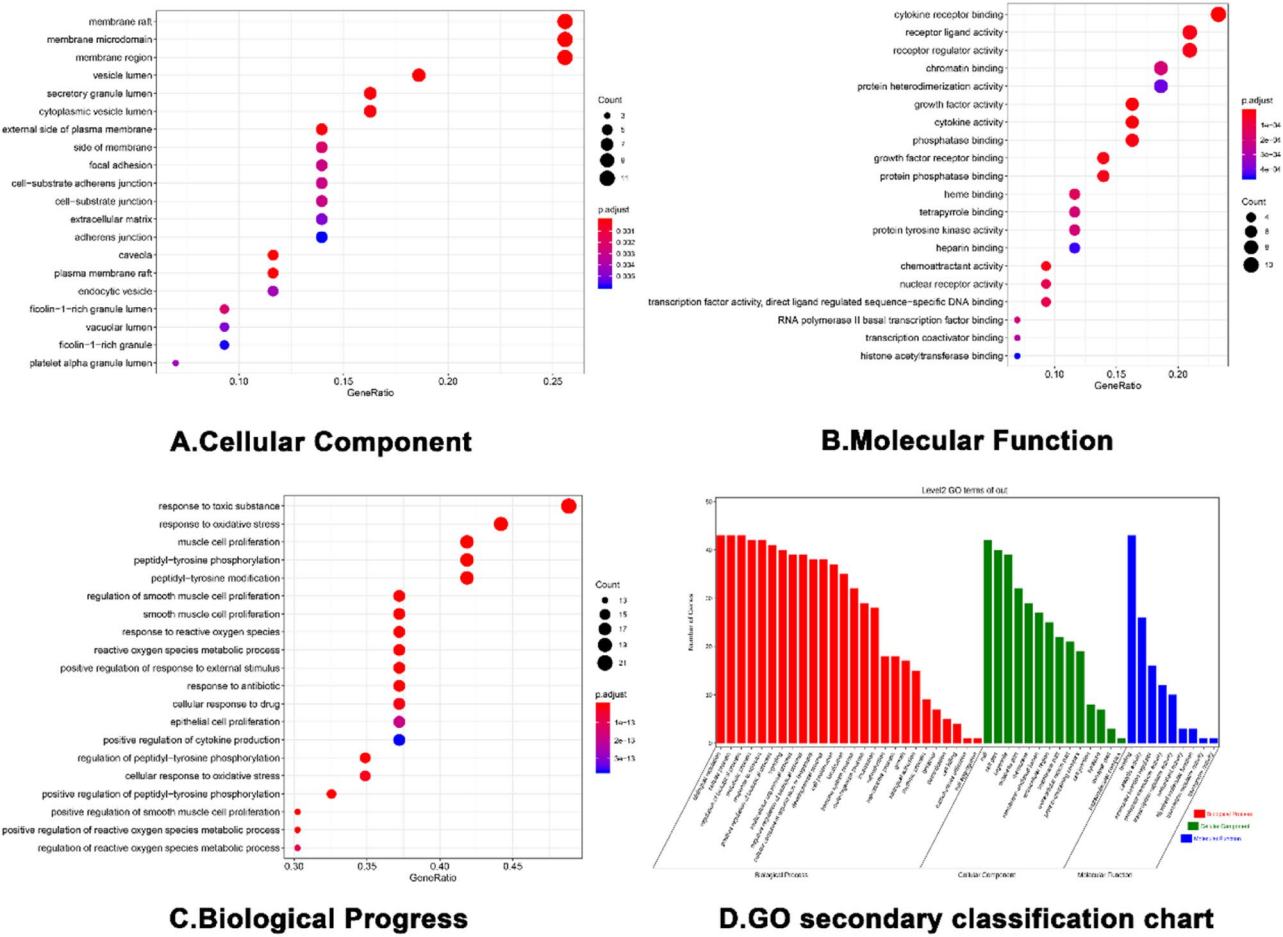
The GO enrichment analysis of core nodes. Including cellular components, molecular functions, biological processes and GO secondary classification.

**Figure 9:**
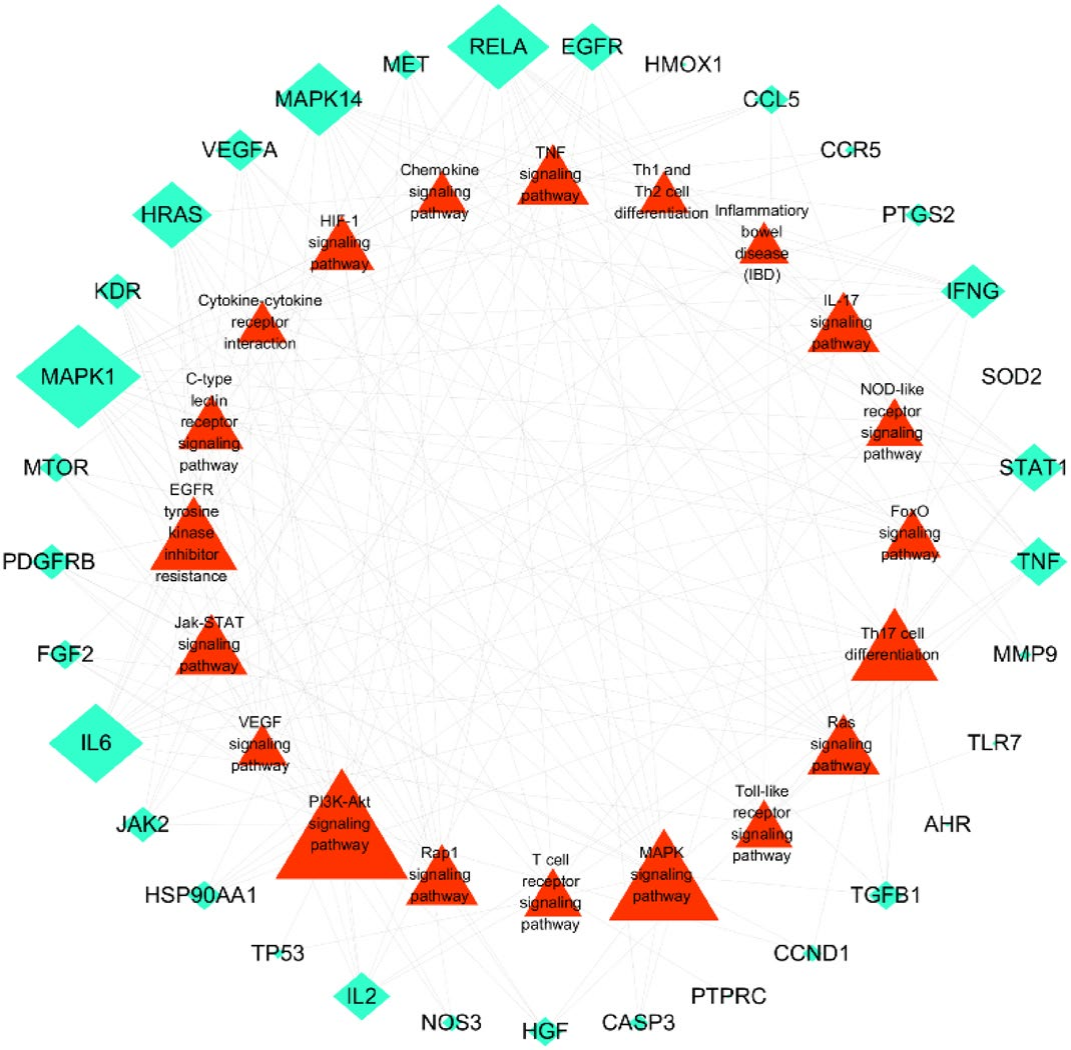
Targets-pathways network (T-P network). The green Rhombus nodes represent the targets of IN-UC. The red triangles represent the related pathways. Node size is relative to degree.

GO enrichment analysis identified 43 core targets involved in cellular composition, molecular function, and biological process. In terms of molecular function, IN treatment of UC mainly involves the regulation of nuclear transcription, protein phosphorylation, cytokine activity and so on, such as cytokine activity (GO:0005125), nuclear receptor activity (GO:0004879), RNA polymerase II basal transcription factor binding (GO:0001091), protein tyrosine kinase activity (GO:0004713). In the biological process mainly involves the modification and metabolic regulation of reactive oxygen species, the positive regulation of epithelial cell proliferation and the participation of apoptosis, such as cell proliferation (GO:0008283), immune system process (GO:0002376), antioxidant activity (GO:0016209), transcription regulator activity (GO:0140110), and translation regulator activity (GO:0045182). At present, IN has been proved to promote the proliferation of intestinal epithelial cells of UC^45^, also promote the secretion of anti-inflammatory factors IL-22 and IL-10, and then repair mucous membrane. At the same time, Indigo and Indirubin have also been proved to have antioxidant activity^46,47^, which is consistent with the conclusions related to MF and BP of GO. (Supplementary Table S7, S8, S9)We selected the first 20 functional enrichment processes to draw a bubble diagram and a secondary classification chart, as shown in Fig. 8. In addition, we identified the main signaling pathways involved in the treatment of UC by KEGG enrichment analysis, and screened the first 20 pathways related to UC and significantly enriched (FDR < 0.05), including PI3K-Akt signaling pathway (hsa04151), MAPK signaling pathway (hsa04010), Th17 cell differentiation (hsa04659), IL-17 signaling pathway (hsa04657), TNF signaling pathway (hsa04668), toll-like receptor signaling pathway (hsa04620), and IBD (hsa05321) among others. The network diagram of “Core targets-Signal pathways” (Fig. 9) was constructed (Supplementary Table S10).

### Components-targets docking analysis

Analysis of the literature and the current research hotspots was performed, and the three components with the highest content of indigo were selected for molecular docking verification, namely indigo, indirubin, and tryptanthrin. AHR, MAPK1, and EGFR were selected from the core targets to download the crystal structures of 5NJ8 (AHR), 5K4I (MAPK1, containing ligands), and 3W2S (EGFR, containing ligands) from RCSB protein data. Since RCSB did not find the effective crystal structure of the AHR binding ligand, and all three are recognized AHR ligands, direct docking was performed with grid center 6.359 30.434 216.222 and NPTS 40 40 40 0.375.

**Figure 10:**
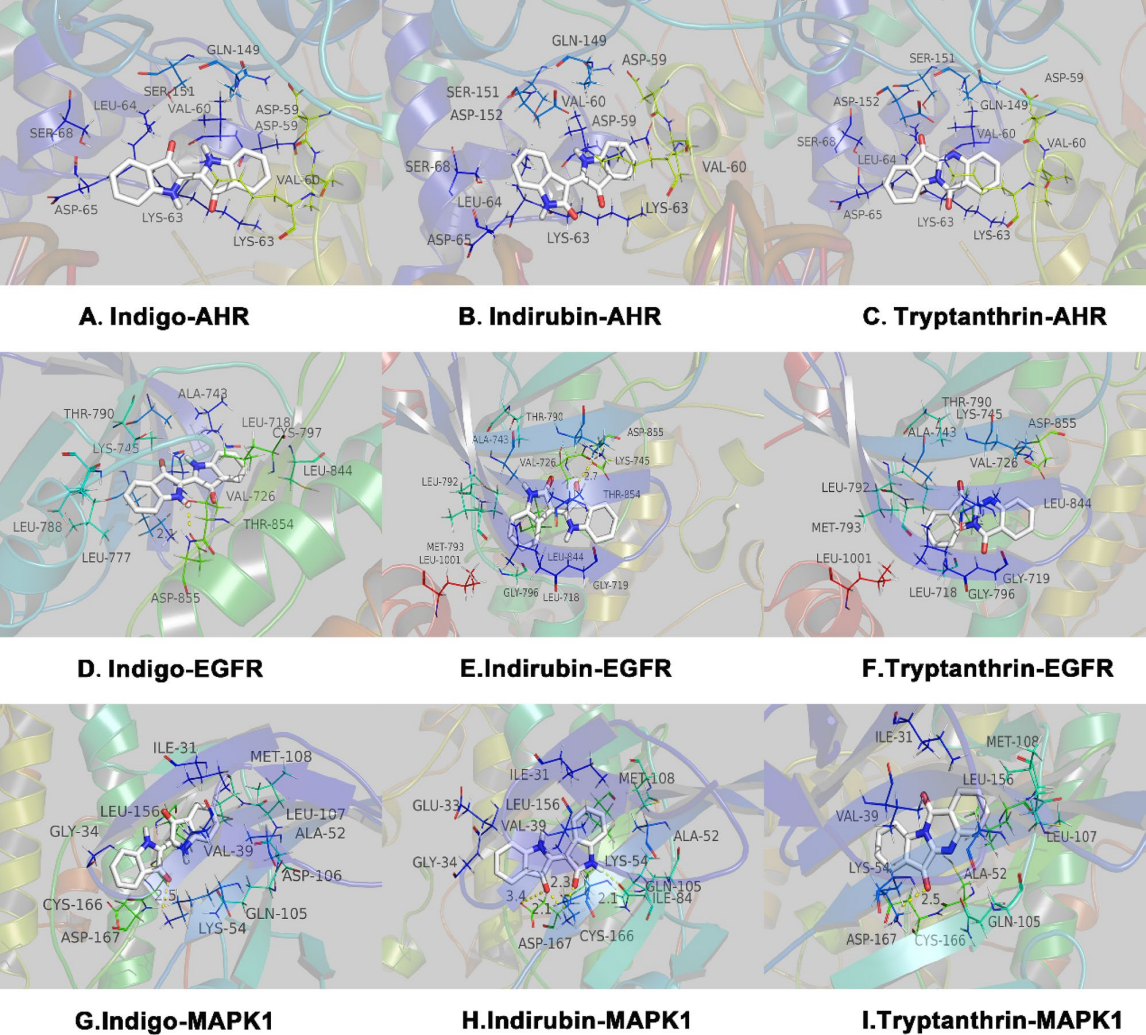
The protein–ligand of the docking simulation. The three core compounds (indigo, indirubin, tryptamethrin) of IN are docked with three targets.

The affinity energy of best mode Indigo-AHR, Indirubin-AHR and Tryptamethrin-AHR are − 6.2 kcal/mol, − 6.7 kcal/mol and − 6.7 kcal/mol. (Supplementary Table S11, S12, S13) The results of indigo-AHR molecular docking showed that both sides of the indole ring have cation-π and anion-π interactions formed by lysine residues (LYS-63) and aspartic acid residues (ASP-65), respectively, and the right indole rings are located in the hydrophobic cavity formed by two valine residues (VAL60). Indirubin-AHR and tryptanthrin-AHR showed the same results. In the process of docking with EGFR, the affinity energy of best mode Indigo-EGFR, Indirubin-EGFR and Tryptamethrin-EGFR are − 8.1 kcal/mol, − 8.5 kcal/mol and − 7.9 kcal/mol.(Supplementary Table S14, S15, S16)The center of the grid is 3.88 1.496 10.744 and the left indole ring of indigo is located in the hydrophobic cavity formed by two leucine residues (LEU-788 and LEU-777). The left indole ring of indigo forms a hydrogen bond with an aspartic acid residue (ASP-855) with an anion-π interaction and a cation-π interaction with a lysine residue (LYS-745). The right indole ring is located in the hydrophobic cavity formed by an alanine residue (ALA-743), a valine residue (VAL-726), and a leucine residue (LEU-718, 844). The left indole ring of indirubin-EGFR and tryptanthrin-EGFR is surrounded by a hydrophobic cavity formed by a hydrophobic alanine (ALA-743), a valine residue (VAL-726), a leucine residue (LEU792, LEU-1001), and a methionine residue (MET-793). The right indole ring forms a cation-π interaction with a lysine residue (LYS-745) and an anion π interaction with an aspartic acid residue (ASP-855). Indirubin also forms a 2.7 Å hydrogen bond with LYS-745. In the process of docking with MAPK1, the affinity energy of best mode Indigo-MAPK1, Indirubin-MAPK1 and Tryptamethrin-MAPK1 are − 8.4 kcal/mol, − 8.8 kcal/mol and − 8.8 kcal/mol. (Supplementary Table S13) The grid center is 14.696 − 4.135 15.275 and the NPTs is 404040 0.375. The left indole ring of indigo and tryptanthrin has 2.5 Å hydrogen bond and a cation-π interaction with a lysine residue (LYS-54), as well as an anion-π interaction with an aspartic acid residue (ASP-167). The right indole ring is surrounded by a hydrophobic lumen formed by an alanine residue (ALA52), a valine residue (VAL-39), a leucine residue (LEU-107, LEU-156), an isoleucine residue (ILE-156), and a methionine residue (MET-108), and it interacts with the anion-π of an aspartic acid residue (ASP-106). The left and right indoles of indirubin-MAPK1 form hydrogen bonds with a lysine residue (LYS-54) at 2.1 Å and 2.3 Å, and the left indole ring also forms a 3.4 Å hydrogen bond with the aspartic acid residue (ASP-167) (Fig. 10).

## Discussion

IN, also known as Qingdai, is a plant derived product commonly used as a dye and pigment. The history of indigo use in China begins with the Theory of Medicinal Materia Medica in the Tang Dynasty. It is used as for heat-clearing and detoxification, cooling blood, and eliminating spots. Clinical formulations contain 1.5–3 g in pills or for external use. In ancient times, it was often used for the treatment of febrile diseases, such as febrile fever, macula, hemoptysis, infantile seizures, sores, erysipelas, and eczema. It has a good curative effect. According to the 2015 edition of Chinese Pharmacopoeia, the authentic source plants of indigo are Malan, Polygonaceae, or Isatis indigotica^48^. It is currently widely used for the treatment of infectious diseases, skin diseases, digestive tract diseases, tumors, and other diseases. IN preparations with evidence-based clinical efficacy are mostly used for psoriasis^49^, leukemia^50^, and UC^51–53^.

Because traditional Chinese medicine is based on a complex system of multiple components, multiple targets and multi-action pathways, the material basis and mechanism of action of its components remain unclear. Therefore, it brings difficulties to the modern research of traditional Chinese medicine. In 2007, Professor Hopkins and Professor Shao Li proposed the concept of network pharmacology and the framework of network pharmacology of traditional Chinese medicine^54^. Network pharmacology of traditional Chinese medicine is a bioinformation network construction and network topology analysis strategy based on high-throughput data analysis, virtual computing, and network database retrieval. The integrity and systematic characteristics of the research strategy of network pharmacology of traditional Chinese medicine are consistent with the principles of diagnosis and treatment of diseases, as well as the synergistic effects of multiple components, multiple pathways, and multiple targets of traditional Chinese medicine and its prescriptions. With the rise of the network pharmacology of traditional Chinese medicine, in the past 10 years, many Chinese medicine scholars have gradually adopted this method to analyze and explore the classical traditional Chinese medicine prescription, single herb and the compounds of traditional Chinese medicine^55,56^. At the same time, the introduction of the concept of network biology revealed that a healthy human body is a dynamically balanced biological network formed by genes, proteins, and other components. If the balance of this network is destroyed, the body presents a state of disease. The objective of the treatment of diseases using drugs is to reconstruct the equilibrium of the biological network or to reduce the degree to which the balance is destroyed based on the ideas described above. Understanding the interactions between drugs and the body and guiding the discovery of new drugs facilitates the treatment of many multi-factor and multi-gene chronic diseases including UC. Many clinical studies have confirmed that prescriptions containing IN are effective for the treatment of UC; therefore, it is important to investigate the mechanism of action of IN and to use the pharmacology of traditional Chinese medicine network for predictive analysis.

In this study, we identified the active components of IN using three databases: TCMSP, TCMID, and BATMAN-TCM. Screening of ADME and related literature led to the identification of ten core active components. Among these ten components, four components (indigo, indirubin, tryptanthrin, and β-sitosterol) were tested in experimental studies using the UC model in vivo and in vitro, and these studies showed different degrees of anti-inflammatory effects. For example, in a UC model using dextran sodium sulfate (DSS) and 6-trinitrobenzenesulfonic acid (TNBS) mice, indigo significantly decreased the severity of colitis. Treatment with indigo significantly increased the levels of CYP1A1, IL-10, and IL-22mRNA in lymphocytes in the lamina propria of the colon. In spleen cells treated with indigo, the number of IL-10 producing CD4 + T cells and IL-22 producing CD3-RoRγ + T cells increased^45^. Indirubin and tryptanthrin are the other two main components of IN. Animal experiments show that indirubin can significantly inhibit DSS-induced weight loss in male and female UC BALB/c mice, reduce the disease activity index), improve colon length and pathological changes, downregulate the expression of the pro-inflammatory factors TNF-α, IL-6, IFN-γ, COX-2, iNOS, PGE2, and NO, and decrease myeloperoxidase (MPO) activity. In addition, it increases the level of IL-10, inhibits the activation of MAPK and NF-κB pathways induced by DSS, upregulates Foxp3, and inhibits the infiltration of CD4 + T cells. Tryptanthrin also improves the body weight and pathology of DSS mice and increases the survival rate. Indirubin regulates the TNF-α/NF-κB p65 and IL-6/STAT3 signaling pathways by inhibiting the degradation of IαBκ and the phosphorylation of STAT3^57,58^. Tryptanthrin also improves the body weight and pathology of DSS mice and increases the survival rate. It regulates the TNF-α/NF-κB p65 and IL-6/STAT3 signaling pathways by inhibiting the degradation of IαBκ and the phosphorylation of STAT3^22^. Spleen cells from mice with colitis treated with tryptanthrin produce less IL-2 and IFN-γ after mitogen stimulation than those from untreated mice. An inflammatory model of RAW264.7 cell UC induced by LPS confirmed that indirubin and tryptanthrin have anti-inflammatory effects in vitro; the anti-inflammatory mechanism may involve the downregulation of IL-6/TNF-α, which can be used for the prevention and treatment of UC^59^.

A common sterol in Chinese herbal medicine, β sitosterol, prevents the shortening of the colon in C57BL/6 mice with colitis induced by TNBS, decreases the general score and myeloperoxidase activity, downregulates the proinflammatory cytokines TNF-α, IL-1β, and IL-6, and the inflammatory enzyme cyclooxygenase (COX)-2, and inhibits the activation of NF-κB^60^. It also significantly increases the expression of antimicrobial peptides in intestinal epithelial cells^61^. Therefore, β-sitosterol may alleviate colitis by inhibiting the NF-κB pathway. Similar results were obtained in C57BL/6 J mice with colitis induced by a high-fat western diet + DSS treated with β-sitosterol^62^. Several active components of IN are effective against UC.

According to the above active components, we performed target fishing using the SEA, STITCH, STP, and PM databases, eliminated repeat compounds, and obtained a total of 933 indigo targets to construct a “drug-component-target” network. Then, we integrated four gene chips of GEO and UC disease targets from DrugBank, DisGeNET, and TTD databases, eliminated duplicates, and finally obtained 913 disease targets. These were matched and mapped to obtain 184 composite IN-UC targets. Then, we constructed a PPI network of composite targets, screened nodes according to the three parameters of DC, BC, and CC, and obtained 43 core target networks to verify the reliability of IN component targets. Three main components of IN were selected to dock with three of the targets, and the docking results were successful according to intermolecular interactions (see the results for specific evaluation criteria). Although the metabolism and effects of oral administration of IN in patients with UC *n vivo* are not clear, we know that IN activates the AHR receptor on type 3 lymphocytes (ILC3) in the lamina propria of the colon, promotes the secretion of the anti-inflammatory factor IL-22, and upregulates regenerated islet-derived REG-1 and REG-3 g to promote the healthy intestinal mucosal barrier^45^. However, the mRNA expression levels of IL-10, IL-22, and Cyp1a1 do not differ between the mesenteric lymphoid node and spleen of DSS C57BL/6 mice and the blank control group. One possible explanation is that IN functions through the local binding of immune cells directly covered by absorbed or unabsorbed IN in the intestinal mucosal surface and intestinal mucosal lamina propria, whereas it does not affect distant immune-related organs. Indirect experiments show that in a 3.5% DSS SD rat model, IN decreases the activity of MPO in the colon, decreases IL1β, IL-18, EGF, and VEGF in serum and colon tissue, and increases the content of the tight junction protein occludin in the colonic mucosal^63^. In a 3% DSS Kunming mouse model, IN significantly downregulated the expression of the pro-inflammatory cytokines IL-6, IL-8, and TNF-α, and upregulated the expression of the anti-inflammatory factor IL10. Combination 16 s rDNA sequence analysis showed that IN decreases the relative number of *Turicibacter bacilli* and increases the relative number of *Peptococcus*^64^*.* In RAW264.7 cells induced by LPS, IN inhibits the degradation of IκB-α, the production of TNF-α and IL-6, and the expression of COX-2 and iNOS^65^.

To explore the underlying mechanism of IN, we performed GO and KEGG enrichment analyses of the core target and retrieved BioGPS to observe the distribution of all compound targets in organs and tissues. GO and KEGG enrichment analyses showed that the core target was enriched in biological process, cellular composition, and molecular function, with a total of 28 items in cellular composition. The targets involved plasma membrane components, cytoplasmic components, and cell junctions, and were enriched to 100 items in molecular functions, which were mainly related to the regulation of nuclear transcription, protein phosphorylation, and cytokine activity among others. In the biological process, it mainly involved the modification and metabolic regulation of reactive oxygen species, the positive regulation of epithelial cell proliferation, and involvement in apoptosis. In addition, we observed 20 pathways related to UC and constructed a “Targets-Pathways” network, which involved innate immunity, cellular immunity, classical inflammation, cell proliferation, and apoptosis. Intestinal innate immunity has recently received increasing attention. UC intestinal immune inflammatory damage is closely related to the abnormal activation of innate immunity, and pattern recognition receptor (PRRs) play an important role in innate immunity. Several types of PRRs (including Toll-like receptors and NOD-like receptors) maintain the mucosal interactive immunity of the intestinal flora by recognizing pathogen-related molecular patterns. Overactivation of TLRs (such as TLR2 and TLR4) and NLRs (such as NLRP3) leads to the activation of NF-κ B, which promotes the production and release of pro-inflammatory factors such as IL-1β, IL-18, and IL-33, and induces the occurrence of cell pyrogenesis^66,67^. The inflammatory necrosis of intestinal epithelial cells and the increase of intestinal mucosal permeability eventually lead to the occurrence and development of UC. For example, Th17 cell differentiation and Jak-STAT, T cell receptor, and IL-17 signaling pathways play an important role in maintaining the balance between Th17 and Treg cells. The immunomodulatory mechanism of Th17 and Tregs has become a hot topic in immunology. Studies show that the pathogenesis of UC is closely related to the imbalance of Th17/Treg^68^. Zhang et al.^69^ showed that the differentiation of Th17 and the expression levels of RORγt, IL-17A, and IL-6 were increased in UC model mice, whereas the differentiation of Tregs and the expression levels of Foxp3 and IL-10 decreased. IL-6 is considered a key factor leading to the imbalance of Th17/Treg differentiation,Kimura et al.^70^ found that AHR ligands (TCDD or FICZ) alone cannot induce the differentiation of Th17 and Tregs. The cytokines IL-6 and TGF-β promote the effect of AHR ligands on increasing the differentiation of Th17 and secretion of IL-17, whereas only TGF-β can increase the expression of Foxp3. Studies also show that different AHR ligands have different effects on inducing Th17/Treg differentiation. The “mirror cells” of CD4 + Th cells-intrinsic lymphocytes (ILCs) were identified as a new target for UC therapy, and were divided into different groups according to the expression of transcription factors, namely, receiving and secreting cytokines including ILC1, ILC2, ILC3, and ILCreg. These localize to barrier tissues and participate in the maintenance of mucosal dynamic balance and host defenses against infection. Japanese scholars showed that indigo, the main ingredient, activates AHR, which upregulates ILC3/IL22 to achieve anti-inflammatory effects. In addition, studies show that ILC2 levels are high in AHR receptors, although AHR inhibits ILC2 function^71^. Among different ILC subsets of the intestine, only ILC2 can be induced to produce IL-10^72^. An ILC regulatory subgroup (also known as ILCreg) secreting IL-10 has not been identified^73^; therefore, how indigo and its main components regulate Th17/Treg and ILCs needs to be further investigated. We found that IN targets were distributed in 84 tissues and organs in the BioGPS database (including colon and small intestine). We selected 17 tissues and organs highly related to immunity (bone marrow, lymph nodes, lymphocytes) to construct an IN targets-organs location network, and most of the targets were highly expressed in several organs. This suggests that IN not only plays a role in the local intestinal mucosa, but it can also stimulate anti-inflammatory processes in distant immune tissues and organs. Thus, IN may also lead to the activation of these targets, inducing right coloitis, pulmonary hypertension, and liver damage. Liver damage associated with IN and the side effects of inducing right colitis and pulmonary hypertension also have this biological basis.

It is known that the network pharmacology of traditional Chinese medicine is a research method aimed at elucidating the effective components and action targets of traditional Chinese medicine, but there is still a lot of room for improvement in the discipline itself. for example, we need to establish more high-quality comprehensive network pharmacology platform. we should fully integrate the existing traditional Chinese medicine, ingredients, syndromes, diseases, targets and other contents, and constantly improve and supplement. At the same time, as traditional Chinese medicine may play a role in the treatment of diseases through the coordination of multiple components, how to predict and evaluate the synergistic effect of multiple compounds is also a challenge we are facing at present. in addition, there is also a lack of information about active components activating or inhibiting targets and signal pathways in the platform. If we can constantly improve the above shortcomings, we will certainly be able to provide a more reliable theoretical basis for the research of traditional Chinese medicine. With regard to the limitations of this manuscript and the aspects that need to be further studied, I think first of all, we can use LC/MS technology to verify and supplement the effective compounds of IN, and carry out corresponding pharmacokinetic and metabonomic studies. In terms of data collection, we can further search other disease databases to supplement disease targets, and verify differential genes combined with patients' colonic mucosa samples. In addition, we also need to use recognized animal and cell models to verify the relevant signal pathways and targets.

## Conclusions

Although IN is an effective mucosal repair agent, the optimal dose for inducing remision with low toxicity needs to be determined. IN is a herb which can eliminate pathogenic factors of body in traditional Chinese medicine; however, whether it is suitable for long-term maintenance treatment of UC remains to be determined in future clinical trials. In addition, basic experiments are necessary to clarify the mechanism of action of this drug.

## Supplementary information

Supplementary Information 1

Supplementary Table 1

Supplementary Table 2

Supplementary Table 3

Supplementary Table 4

Supplementary Table 5

Supplementary Table 6

Supplementary Table 7

Supplementary Table 8

Supplementary Table 9

Supplementary Table 10

Supplementary Table 11

Supplementary Table 12

Supplementary Table 13

Supplementary Table 14

Supplementary Table 15

Supplementary Table 16

Supplementary Table 17

Supplementary Table 18

Supplementary Table 19

## Data Availability

All the data can be obtained from the open source website we provide, and the conclusion can be drawn through the analysis of the relevant software.
