# Supplementary material for: Mechanisms of indigo naturalis on treating ulcerative colitis explored by GEO gene chips combined with network pharmacology and molecular docking
Source: Sci Rep. 2020 Sep 16;10:15204. doi: 10.1038/s41598-020-71030-w (PMC7495487; doi:10.1038/s41598-020-71030-w)
Supplement: Supplementary file 1 — Supplementary Information 1 [file 41598_2020_71030_MOESM1_ESM.docx]

**Mechanisms of Indigo Naturalis on treating ulcerative colitis explored by GEO gene chips combined with network pharmacology and molecular docking**

**Sizhen Gu^1^, Yan Xue^2^, Yang Gao^1^, Shuyang Shen^1^, Yuli Zhang^1^, Kanjun Chen^1^, Shigui Xue^3^, Ji Pan^3^, Yini Tang^3^, Hui Zhu ^4^, Huan Wu^1^, Danbo Dou*^1^**



 10h-indolo[3,2-b]quinoline



 beta-Sitosterol



 Bisindigotin



 Indican



 indigo



 Indirubin



 Isoindigo



 Isovitexin



 Qingdainone



 tryptanthrin
